# A Case of Transient Complete Heart Block During Left Heart Catheterization

**DOI:** 10.7759/cureus.62161

**Published:** 2024-06-11

**Authors:** Sushant Koirala, Raed Qarajeh, Fareed Collado

**Affiliations:** 1 Internal Medicine, Rush University Medical Center, Chicago, USA; 2 Medicine/Cardiology, Rush University Medical Center, Chicago, USA

**Keywords:** left heart catheterization, intraprocedure complication, iatrogenic transient heart block, aortic valve disease, cardiac electrophysiology

## Abstract

Iatrogenic complete heart blocks are rare but a reported complication of left heart catheterizations in patients with pre-existing right bundle branch blocks. We present the case of an 84-year-old male with a preexisting right bundle branch block who underwent a left heart catheterization for valve replacement evaluation. While attempting to engage the right coronary artery, the catheter instead crossed the aortic valve, causing the patient to become bradycardic to the 20s and hypotensive. The patient had a temporary transvenous pacer inserted and tolerated the rest of the procedure well. The cause of the complete heart block was thought to be due to the transient blockage of the left bundle branch due to ventricular septal irritation when the catheter crossed the aortic valve. When performing left heart angiograms in a patient with a right bundle branch block, operators should be prepared for a possible iatrogenic complete heart block.

## Introduction

Due to the proximity of the left bundle branch to the aortic valve, complete heart blocks (CHBs) or high-degree atrioventricular blocks are a known risk for patients with pre-existing right bundle branch blocks (RBBBs) undergoing transcatheter aortic valve replacement (TAVR) [1]. Studies have shown that those with reported RBBBs have up to a 26-fold increase in the risk of having a delayed onset of arrhythmia [2]. Less common, however, is a CHB seen in patients undergoing left heart catheterization (LHC). We present the case of a patient undergoing LHC as part of his TAVR workup which was complicated by an intra-procedure CHB.

## Case presentation

**Figure 1:**
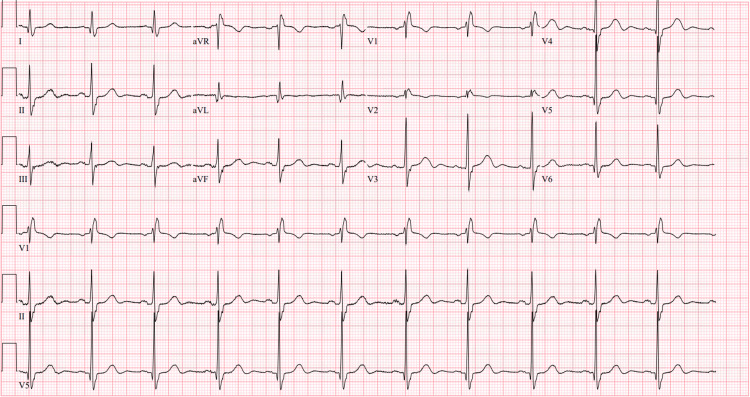
Electrocardiogram showing a right bundle branch block before left heart catheterization.

An 84-year-old man presented for a coronary angiogram as part of a pre-TAVR evaluation. The patient had a history of severe aortic stenosis (mean gradient 44 mmHg, peak velocity 4.1 m/s, and aortic valve area of 0.9 cm²), known previous RBBB (Figure 1), hypertension, dyslipidemia, hypothyroidism, stage I colorectal cancer s/p partial colectomy, and supraventricular tachycardia s/p radiofrequency atrioventricular nodal reentrant tachycardia ablation.

**Figure 2:**
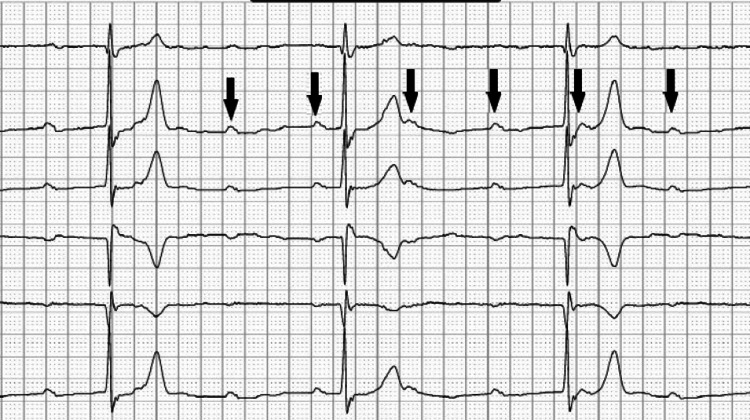
Complete heart block captured on telemetry during left heart catheterization. The arrows point to the non-conducting P waves.

**Figure 3:**
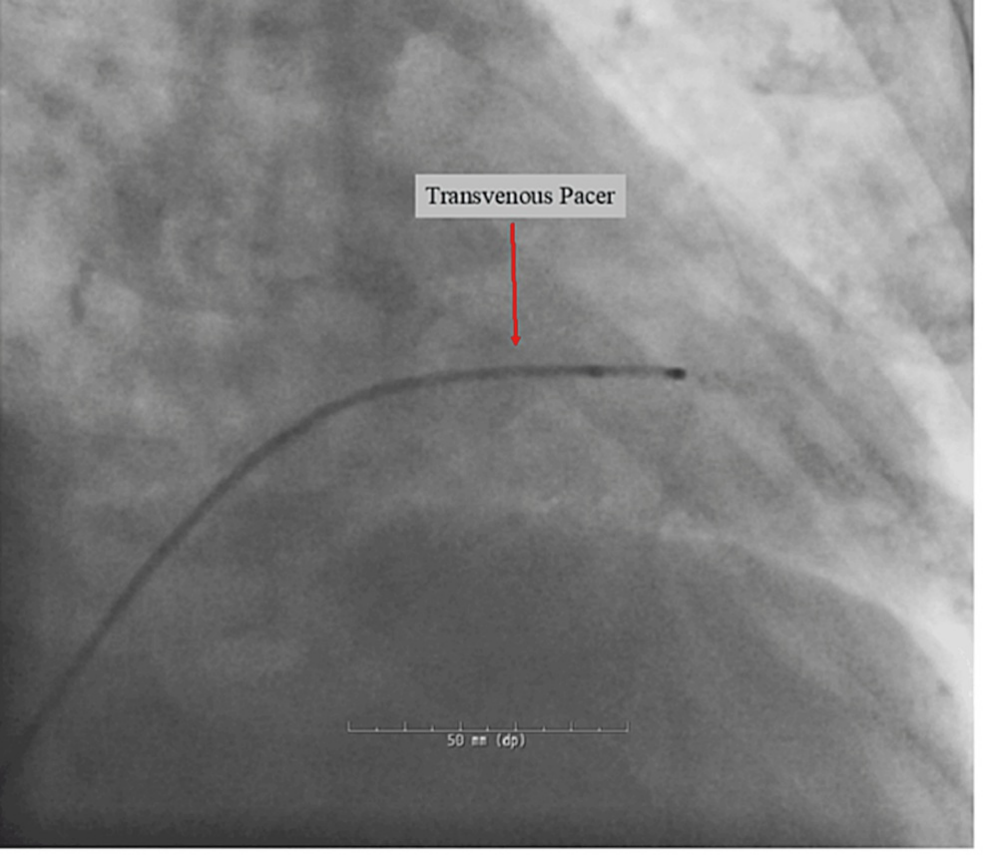
Transvenous pacer.

A baseline electrocardiogram (EKG) was recorded along with basic vitals before the coronary angiogram. The baseline EKG showed a normal sinus rhythm with the previously known RBBB. The right radial artery was accessed for the procedure. Selective left coronary angiography was performed using a Radial TIG 4.0 catheter and images were obtained using multiple projections. A diffuse 30% stenosis was seen in the left main coronary artery with a mild diffuse disease of the left anterior descending (LAD) artery with a discrete 70% stenosis of the proximal LAD. The left circumflex had a proximal discrete 40% stenosis. A selective right coronary angiography was attempted next using a Judkins Right 4.0 (JR 4.0) catheter. While trying to engage the right coronary artery (RCA), the catheter crossed the aortic valve, and the patient became bradycardic to a heart rate in the 20s to 30s beats per minute with continuous cardiac monitoring consistent with CHB (Figure 2). The patient became hypotensive requiring medical therapy with atropine, dopamine infusion, and a transvenous temporary pacer was inserted (Figure 3). Transient CHB and blood pressure improved, and we were able to wean off dopamine infusion. Continuous cardiac monitoring showed restoration of intrinsic normal sinus rhythm and the temporary pacer was set at 40 beats-per-minute backup rate. Right coronary angiography showed no disease in the RCA, but the right posterior descending artery had proximal 60% diffuse stenosis. The patient was then admitted to the cardiovascular intensive care unit for further monitoring.

## Discussion

Although CHB is a commonly seen complication in patients with preexisting RBBB undergoing TAVR, the number of cases of reported LHC cases being complicated by CHB is significantly more scarce [3]. The few case reports showing this phenomenon were with patients with preexisting RBBB, as seen in our patient, and in each of them, the proceduralist crossed the aortic valve and entered the left ventricle [4,5]. Other similar cases have also been reported of a patient with a left bundle branch block who had a pulmonary catheterization or other right-sided catheterizations [6,7]. What these cases have in common is a patient with preexisting conduction abnormality undergoing a procedure that causes an iatrogenic irritation of the membranous septum or ventricular septum, causing a transient CHB due to irritation of the previously functional bundle branch [8,9]. The His bundle, as it comes off the atrioventricular node, lies adjacent to the membranous septum. Furthermore, as the His bundle separated into the right bundle and left bundle branches, the left bundle branch originates close to the right coronary and non-coronary cusps of the aortic valve leaflets. As such, it is likely that in the general population without a preexisting RBBB transient iatrogenic irritation of the membranous septum is unlikely to cause a CHB.

**Figure 4:**
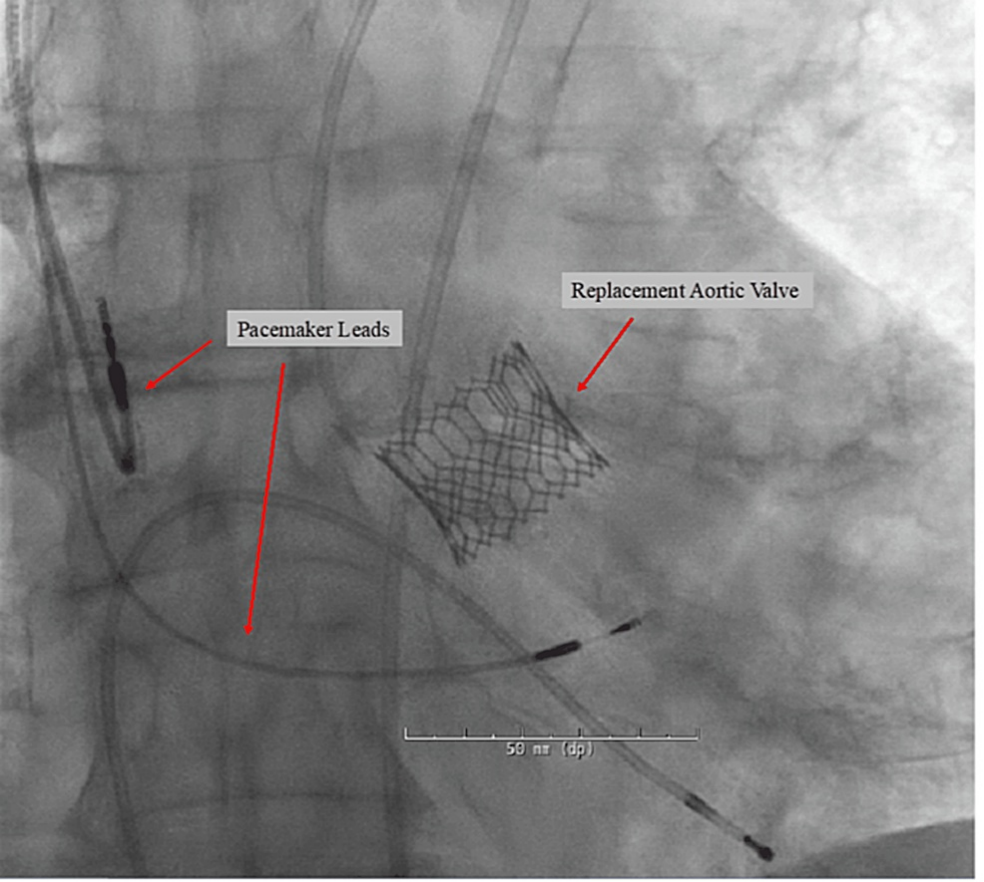
Status post-transcatheter aortic valve replacement and permanent pacemaker placement.

In the case of our patient, the pathophysiology of the heart block can be explained by the proximity of the heart’s conduction system to the aortic valve leaflets. We cannot be certain as to what specifically caused the CHB in our patient due to his prior RBBB, as both the trauma of the membranous septum, causing irritation of the bundle of His, or trauma to the muscular interventricular septum, causing irritation of the functioning left bundle branch would cause similar outcomes of a CHB. Despite the volume of LHC completed, the rarity of this complication can be explained simply by the rarity of RBBB. According to the American Heart Association, RBBB occurs in only 0.2%-1.2% of the population [10]. Moreover, as TAVRs become more common, iatrogenic CHB secondary to LHC becomes more important to consider minimizing the risk of a post-TAVR delayed conduction delay. In this case, the induced CHB led the cardiac team to pre-emptively place a permanent pacemaker before the TAVR. The patient later underwent TAVR and tolerated both procedures well (Figure 4).

## Conclusions

CHB is an observed complication of many procedures in the catheterization lab. However, even LHC without intervention can cause CHB in patients with prior RBBB. During LHC in patients with a baseline conduction system disease, transient CHB can occur upon crossing the aortic valve and operators should be prepared for this potential complication. The rarity of this phenomenon is likely due to the rarity of RBBB.
